# Mortality Table

**Published:** 1879-11

**Authors:** 


### MORTALITY TABLE.

CONDENSED from National Board of Health Bulletin for the four weeks ending Sept. 27th, 1879.

| CITIES. | Estimated Population. | Deaths. | Death rate per 1000. |
|---|---|---|---|
| BALTIMORE, - - - - - - | 400.000 | 557 | 18.10 |
| BOSTON, - - - - - - | 365,000 | 514 | 18.30 |
| **BUFFALO,** - - - - | **170,000** | **113** | **7.97** |
| CHICAGO, - - - - - - | 460,000 | 634 | 17.92 |
| CLEVELAND, - - - - - - | 160,000 | 189 | 15.36 |
| LOUISVILLE, - - - - - - | 175,000 | 223 | 16.54 |
| NEW YORK, - - - - - - | 1,097,000 | 2025 | 23.00 |
| PHILADELPHIA, - - - - | 901,000 | 1099 | 15.85 |
| ROCHESTER, - - - - - - | 90,000 | *57 | 16.36 |
| ST. LOUIS, - - - - - - | 500,000 | 424 | 11.02 |

* Only two weeks reported by National Board of Health.

